# Superior Mesenteric Artery Dissection After Lumbar Puncture

**DOI:** 10.7759/cureus.7507

**Published:** 2020-04-02

**Authors:** Luz M Ramirez, Sebastian Casillas, Hussein Berjaoui, Joseph Varon, Salim Surani

**Affiliations:** 1 Pulmonology and Critical Care, Benemerita Universidad Autonoma De Puebla, Puebla, MEX; 2 Medicine, Dorrington Medical Associates, Houston, USA; 3 Medicine, Beirut Arab University, Beirut, LBN; 4 Critical Care, United General Hospital, Houston, USA; 5 Critical Care, University of Texas Health Science Center, Houston, USA; 6 Internal Medicine, Texas A&M Health Science Center, Bryan, USA; 7 Internal Medicine, Corpus Christi Medical Center, Corpus Christi, USA; 8 Internal Medicine, University of North Texas, Dallas, USA

**Keywords:** superior mesenteric artery dissection, lumbar puncture, hemoperitoneum, retroperitoneal bleed, spinal tap

## Abstract

We hereby present a case of iatrogenic dissection of the superior mesenteric artery dissection in a 63-year-old female undergoing a lumbar puncture (LP). She presented with severe diffused abdominal pain accompanied by lower back pain, nausea and vomiting a few hours after undergoing an LP due to ongoing headaches. Abdominal CT showed evidence of hemoperitoneum. She was then transferred to another facility and while in route received one unit of packed red blood cellsdue to drop in hemoglobin levels from 15 to 11 gm/dl. Physicians should consider the possibility of arterial variations and the level at which spinal tap is performed during interventions. Acute abdominal pain is a significant, common complaint that should be appropriately investigated.

## Introduction

Mesenteric vascularity is a rich mixture of vessels that provide the main blood supply to the gastrointestinal tract [1]. It is considered when anterior abdominal procedures are performed. However, when a posterior approach such as a lumbar puncture (LP) or any retroperitoneal procedures are performed, the importance is often overlooked. Superior mesenteric artery (SMA) is a branch of the aorta that arises 1 cm below the celiac trunk, at the level of L1- L2, and typically precedes three colic arteries (middle, right and ileocolic arteries); nevertheless, different patterns have been reported showing the presence of supernumerary arteries and anatomical variations [1-3].

## Case presentation

A 63-year-old female presented to the emergency department (ED) with a four-day history of severe sudden headaches. An LP was then performed with dry tap. Few hours later, the patient complaint of severe diffused abdominal pain accompanied by lower back pain, nausea and vomiting. She denied diarrhea, hematochezia and melena.

Her past medical history was significant for migraine, dyslipidemia, chronic neck pain and chronic back pain. Her previous surgeries included appendectomy, cholecystectomy, oophorectomy and back surgery. She denied any known allergies, use of tobacco or illicit drug abuse, but admitted to occasional use of alcohol.

**Figure 1:**
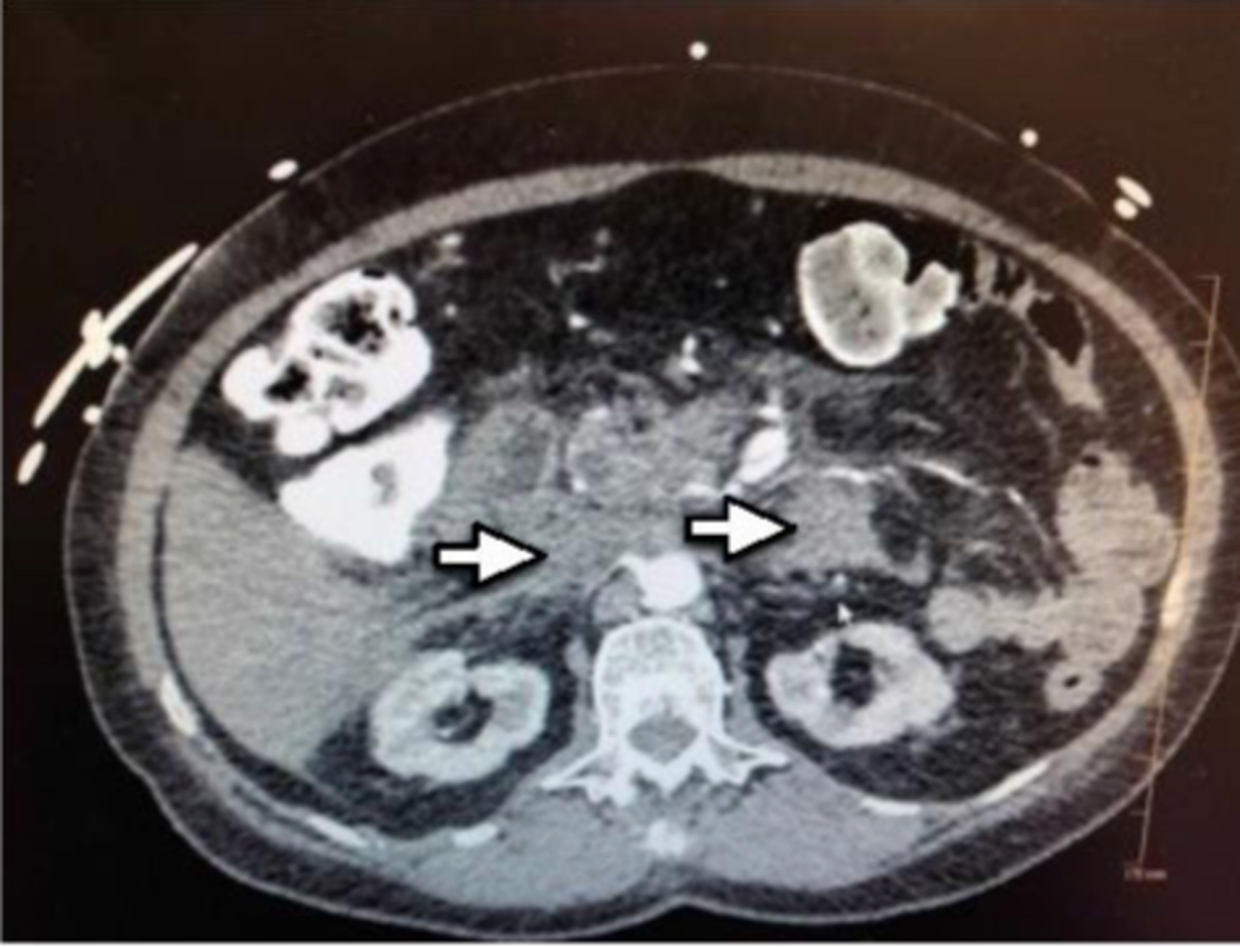
CT of the abdomen showing a diffuse hemorrhage in the peritoneum as indicated by the arrow.

On physical examination, she was afebrile (98.6ºF) with a blood pressure (BP) of 133/70 mmHg, a heart rate of 95 bpm, a respiratory rate of 20 breaths/min and an oxygen saturation of 99% (SpO_2_) at room air. Her body mass index was 24.6 kg/m^2^. She had soft, tender abdomen with normal bowel movements, positive to Blumberg's sign (rebound tenderness). The rest of the physical examination was unremarkable. The patient underwent abdominal CT, which showed evidence of hemoperitoneum (Figure 1). She was then transferred to another facility and while in route received one unit of packed red blood cells was given due to a drop in hemoglobin (Hb) levels from 15 to 11 gm/dl. After transfusion, Hb increased to 12 gm/dl.

At arrival to the our ED, she had a computed tomography angiography (CTA) of the abdomen and pelvis with intravenous (IV) contrast that reported a 2 cm flap of the SMA as the most likely source of bleeding with the root of the mesentery measuring 5 cm x 3 cm transverse. Laboratory tests included the following: complete metabolic panel, with blood urea nitrogen of 23 mg/dl, carbon dioxide level of 22 mEq/l and chloride level of 112 mEq/l; complete blood count with white blood count of 13.9x10^9^/L, hematocrit of 37.6%, Hb of 12 gm/dl and platelet count of 178,000/mm^3^; coagulation profile with prothrombin time of 9.9 seconds, partial thromboplastin time of 20 seconds and international normalized ratio of 1.0.

**Figure 2:**
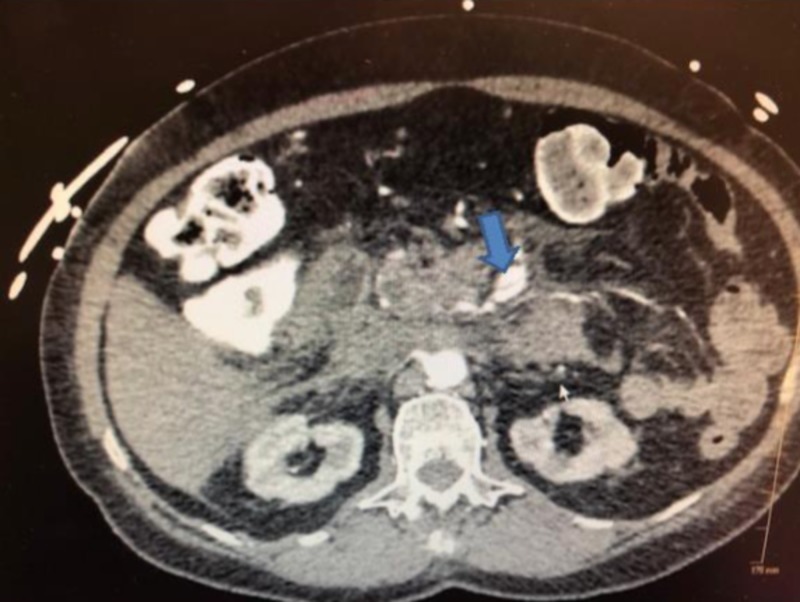
CT scan showing dissection of superior mesenteric artery and intimal flap.

The patient was admitted to the intensive care unit (ICU) for intraperitoneal bleed. A repeated CTA of the abdomen and pelvis with IV contrast after 48 hours showed focal dissection in the SMA originated 2 cm from vessel origin, with a fenestration involving the proximal portion or the dissection and an existing component of the root of the mesentery, measuring 5 cm transverse x 3 cm anteroposterior (AP) (Figure 2). An evolving retroperitoneal hematoma was also seen, measuring 16.1 cm transverse x 5.2 cm AP by about 8.4 craniocaudal, involving the pancreatic head and both third and fourth portions of the duodenum, anterior to Gerota’s fascia on the right, extending into the root of the mesentery and the bilateral paracolic gutters.

Hb and hematocrit levels were monitored, and a management of the systolic BP between 110 and 120 mmHg was made. The patient was transferred to tertiary center where she underwent surgical correction and was discharged home in stable condition.

## Discussion

LP is considered a relatively safe procedure, often served as an important diagnostic tool for a wide range of neurological conditions [4]. With most cases, the exact level of entry of the spinal needle is determined with the patient standing straight or sitting upright at an intersecting line at L1-L2 to L4-L5 range, usually pointing at a higher spinal level in female and obese patients. Shortly after, insertion into the subarachnoid space at the L3-L4 or L4-L5 interspace is made [5].

Therefore, acceptable control measures, precise technique and understanding the normal anatomy and the variants of the vascular network are important in order to avoid iatrogenic complications [6]. The branches of the abdominal aorta where the SMA is located should be taken into consideration.

Commonly known as the artery of the midgut, the SMA sends nearly 15-18 branches, irrigating two out of three portions of the transverse colon and other organs from the abdominal cavity [7]. The pathway goes downwards and to the back of the splenic vein and the thin section of the pancreas. Following, on the right side, in relation with the left renal vein, directing to the uncinate process of the pancreas and then to the anterior third of the duodenum. Ileal branches originated, and soon after they connect with the ileal branch of the ileocolic artery, unless anatomic variations are present [8]. The literature established that anatomic variations of the SMA and its branches are common. In up to 52% of the cases, the middle colic artery and right colic artery can arise from the common trunk. In 38% of the cases, the right colic artery is a separate branch of the SMA and 8% arises in the ileocolic artery [9]. Existence of an accessory right colic branching has also been reported in 8%-10%, as well as the presence of a long branch of the middle colic artery connecting to the splenic flexure [2]. At this point in terms of anatomic variations, trying to find a logic explanation of the artery dissection which is defined as an abrupt and abnormal focal tear in the tunica intima results in the exposure of the deep layers of the artery [10]. As Prabhakar et al previously established, a middle colic artery connecting to the splenic flexure can lead to this event, if anatomically positioned in relation to the possible LP approach we can topographically project a point between this anatomic variant, the spleen and the spinal cord, since the aorta was avoided and the CT scan showed a SMA dissection [2]. Our patient had inadvertently underwent LP at the higher level, which possibly can cause the event; the clinical features that most patients present with SMA dissection are severe abdominal pain associated with nausea, vomiting, abdominal distension and melena [11]. Moreover, other possibility can also be the spontaneous dissection of the SMA [12-15].

Health care providers need to consider the complications of this clinical entity such as ischemia, bowel infarction, acute peritonitis, shock and in chronic cases uremia and intra-abdominal hemorrhage giving all the clinical features that are explained in the case presentation [16].

CT scans in the evaluation of nonspecific abdominal pain have allowed physicians to detect these dissections and treat them accordingly, but in this case the hemorrhage can turn this test difficult to read [17].

## Conclusions

In order to acknowledge a SMA dissection, it is extremely important to consider differential diagnosis and appropriate selection of additional diagnostics tests. Iatrogenic complications can happen in the event of vascular variation if high-level LP is performed and spontaneous dissection needs to be considered. Appropriate vigilance needs to be undertaken in evaluating the patient with abdominal and back pain with drop in hemoglobin.
